# Effect of stochasticity on coinfection dynamics of respiratory viruses

**DOI:** 10.1186/s12859-019-2793-6

**Published:** 2019-04-16

**Authors:** Lubna Pinky, Gilberto Gonzalez-Parra, Hana M. Dobrovolny

**Affiliations:** 1 Department of Physics & Astronomy, Texas Christian University, Fort Worth, TX, USA; 2 Department of Pediatrics, University of Tennessee Health Science Center, Memphis, TN, USA; 3 Department of Mathematics, New Mexico Tech, Socorro, NM, USA

**Keywords:** Viral coinfection, Respiratory virus, Within-host model, Continuous-time Markov chain, Multi-type branching process, Extinction probability

## Abstract

**Background:**

Respiratory viral infections are a leading cause of mortality worldwide. As many as 40% of patients hospitalized with influenza-like illness are reported to be infected with more than one type of virus. However, it is not clear whether these infections are more severe than single viral infections. Mathematical models can be used to help us understand the dynamics of respiratory viral coinfections and their impact on the severity of the illness. Most models of viral infections use ordinary differential equations (ODE) that reproduce the average behavior of the infection, however, they might be inaccurate in predicting certain events because of the stochastic nature of viral replication cycle. Stochastic simulations of single virus infections have shown that there is an extinction probability that depends on the size of the initial viral inoculum and parameters that describe virus-cell interactions. Thus the coinfection dynamics predicted by the ODE might be difficult to observe in reality.

**Results:**

In this work, a continuous-time Markov chain (CTMC) model is formulated to investigate probabilistic outcomes of coinfections. This CTMC model is based on our previous coinfection model, expressed in terms of a system of ordinary differential equations. Using the Gillespie method for stochastic simulation, we examine whether stochastic effects early in the infection can alter which virus dominates the infection.

**Conclusions:**

We derive extinction probabilities for each virus individually as well as for the infection as a whole. We find that unlike the prediction of the ODE model, for similar initial growth rates stochasticity allows for a slower growing virus to out-compete a faster growing virus.

**Electronic supplementary material:**

The online version of this article (10.1186/s12859-019-2793-6) contains supplementary material, which is available to authorized users.

## Background

With the advent of molecular diagnostic techniques, respiratory tract specimens from patients with influenza-like illness (ILI) are now being recognized as having multiple viruses [1–4]. Around 40% of the hospitalized patients with ILI have coinfections with influenza A virus (IAV), influenza B virus (IBV), respiratory syncytial virus (RSV), human rhinovirus (hRV), adenovirus (AdV), human enterovirus (hEV), human metapneumovirus (hMPV), coronavirus (CoV), parainfluenza virus (PIV), human bocavirus (hBoV) and many others [5–9]. These patients are reported to suffer from heterogeneous disease outcomes such as enhanced [10–12], reduced [13, 14] and unaltered [14–16] severity compared to patients with single virus infections. However, it is not clear how the virus-virus and virus-host interactions influence disease severity and lead to these varied outcomes. Two or more virus agents can interact in diverse ways which may arise from the consequences of their inoculation order, inter exposure time, initial inoculums, different combinations of viruses, number of coinfecting viruses and host immune state [17, 18]. Thus, coinfections pose a combinatorial problem which can be challenging to study in a laboratory set up alone.

Coinfection can be better understood using mathematical modeling. While mathematical modeling of single virus infections at the cellular level has proven crucial for finding answers where laboratory experiments are impossible, impractical or expensive [19–23], little has been done in viral coinfection modeling. A few studies [24–26] have used within host models considering interactions of two different strains of the same virus. Among them, Pinilla et al. [24] and Petrie et al. [25] used their models to study competitive mixed-infection experiments of pandemic A/H1N1 influenza with its H275Y mutant strain and Simeonov et al. [26] considered a spatio-temporal model to explain in vitro cellular susceptibility due to the simultaneous presence of RSV A2 and RSV B. Pinky and Dobrovolny [27] proposed a two virus coinfection model to investigate viral interference observed in an experimental study of IAV-RSV coinfection (Shinjoh et al. [28]) where they concluded that distinct viruses interact through resource competition. In further investigations [29, 30], they used the model to quantify the impact of resource availability, finding the possibility of chronic single infection if constant cellular regeneration was considered and chronic coinfection if both cellular regeneration and superinfection were considered. However, the majority of the two virus models studied so far have focused on the deterministic approach that reproduces the average behavior of infection kinetics. The exceptions are Dobrovolny et al. [31] and Deecke et al. [32] who investigated two strains of the same virus (wild-type and drug resistant mutant) using a stochastic model to determine mechanisms driving the emergence of drug-resistant mutants during the course of a single infection. Since in real life viral infections are stochastic and discrete events, stochastic simulations of infection models will provide further insight into coinfection dynamics.

For example, stochastic simulations of single virus infections have shown that there is an extinction probability that depends on the size of the initial viral inoculum and parameters that describe virus-cell interactions [33]. Similarly, experimental studies of viral infections in animals have shown that viruses do not always establish infection in every animal under study [34]. Although the causative phenomenon is still unidentified, there are some possible factors suggested by researchers such as host defense mechanisms, spatial heterogeneity in the target cell population, and the stochastic nature of the virus life cycle [34]. Moreover, evaluation of this quantity can be useful in many situations where the viral dynamics cannot be explained with a simple deterministic model. Numerous stochastic models have been developed to study various aspects of the single viral infection process such as virus release strategies (i.e. budding and bursting) for HIV [33, 34], impact of initial viral dose [35], length of eclipse and infectious phases [33, 34], impact of the immune response [34, 35], and how ongoing proliferation of immune cells acts to decrease the emergence probability of mutated strains [36]. These models have been studied using Monte Carlo simulations of the multi-type branching process [37, 38], or by simulating solutions to stochastic differential equations where processes involved in the virus life cycle are diffusion processes (stochasticity is represented by noise terms in the equations) [35, 39, 40].

Of particular interest for stochastic models is the probability of extinction, a feature that ODE models cannot capture. In stochastic models, analytic expression of extinction probability is formulated by keeping track of the number of infected cells [41], the number of virions [42] and both [33] in single virus models, mostly for HIV infection. Yan et al. [34] used a similar method to calculate the extinction probability that includes time dependent immune responses in a single influenza virus model. Stochastic extinction could be a factor in coinfection dynamics since one virus could have a higher extinction probability, even if the two viruses have the same initial viral inoculum or initial growth rate, making it possible for one virus to go extinct while the other viral infection grows. Thus the coinfection outcomes predicted by the ODE model might be difficult to observe in reality.

In this work, we implement a stochastic counterpart of our previously published ODE coinfection model [27], in the form of a continuous-time Markov chain (CTMC) model. Trajectories for the CTMC model are simulated using Gillespie’s tau-leap algorithm. In order to investigate how stochastic effects early in the infection impact coinfection, we vary the initial growth rate and compare to predictions from the ODE model. We also derive the extinction coefficient analytically for the model using multi-type branching method. While the ODE model found that the virus with a higher growth rate consumes more target cells and produces higher peak viral load compared to the slower growing virus, we find that stochasticity can allow slower growing viruses to consume more target cells and produce more virus than the faster growing virus.

## Results

### Derivation of extinction coefficient

Stochastic extinction is most relevant during the initial stage of infection. At this stage the number of target cells is small. We can consider that the target cells are constant or equal to the initial number of target cells (*T* ≈ *T*_0_). As a result, the states become decoupled making the stochastic events independent of each other. In addition, each event produces progeny over a lifetime which is also independent of the lifetime of all other events. More details on how to derive a branching process from a CTMC can be found in [43]. Under these conditions, the CTMC model becomes a multi-type branching process where the reduced state vectors now represent $\vec {m}$=$(n_{E_{1}}$, $n_{I_{1}}, n_{V_{1}}$, $n_{E_{2}}, n_{I_{2}}, n_{V_{2}})$, where $n_{E_{1}}$ and $n_{E_{2}}$ are the numbers of eclipse cells, $n_{I_{1}}$ and $n_{I_{2}}$ are the infected cells, and $n_{V_{1}}$ and $n_{V_{2}}$ are the virions of both viruses. Including the assumption of a constant number of target cells, the reduced model is

$$\begin{array}{*{20}l} V_{1}\xrightarrow {\beta_{1} T} E_{1} && V_{2}\xrightarrow {\beta_{2} T} E_{2}\\ E_{1}\xrightarrow {k_{1}} I_{1} && E_{2}\xrightarrow {k_{2}} I_{2}\\ I_{1}\xrightarrow {p_{1}} V_{1} && I_{2}\xrightarrow {p_{2}} V_{2}\\ I_{1}\xrightarrow {\delta_{1}} \emptyset && I_{2}\xrightarrow {\delta_{2}} \emptyset \\ V_{1}\xrightarrow {c_{1}} \emptyset && V_{2}\xrightarrow {c_{2}} \emptyset. \\ \end{array} $$

Thus the continuous-time Markov chain becomes a multi-type branching process that describes the dynamics of a population of individuals having birth and death independently according to the specified (in this case exponential) probability mass function. If a time-homogeneous CTMC is a branching process, the only absorbing state is $\vec {0}$. For this model we defined the absorbing state as $\vec {0}$ and the probability to reach this state from, say, $\vec {m}$, is $\xi (\vec {m})$. This probability is referred to as the extinction probability. Biologically, the extinction probability is defined as the probability that the two types of viruses and all their infected cells are completely eliminated from the host. Once a transition occurs, the current state $\vec {m}$ is incremented by one of the transition vectors given below.

$$\begin{array}{*{20}l} d\vec{m_{1}}=(0, +1, 0, 0, 0,0)\ & \text{for}\ V_{1}\xrightarrow {\beta_{1} T} E_{1}\\ d\vec{m_{2}}=(0, -1, +1, 0, 0, 0)\ & \text{for}\ E_{1}\xrightarrow {k_{1}} I_{1} \\ d\vec{m_{3}}=(+1, 0, 0, 0, 0, 0) \ &\text{for} \ I_{1}\xrightarrow {p_{1}} V_{1} \\ d\vec{m_{4}}=(0, 0, -1, 0, 0, 0) \ & \text{for}\ I_{1}\xrightarrow {\delta_{1}} \phi \\ d\vec{m_{5}}=(-1, 0, 0, 0, 0, 0)\ & \text{for}\ V_{1}\xrightarrow {c_{1}} \phi \\ d\vec{m_{6}}=(0, 0, 0, 0, +1, 0)\ & \text{for}\ V_{2}\xrightarrow {\beta_{2} T} E_{2} \\ d\vec{m_{7}}=(0, 0, 0,0, -1, +1)\ & \text{for}\ E_{2}\xrightarrow {k_{2}} I_{2} \\ d\vec{m_{8}}=(0, 0, 0, +1, 0, 0)\ &\text{for} \ I_{2}\xrightarrow {p_{2}} V_{2} \\ d\vec{m_{9}}=(0, 0, 0, 0, 0, -1)\ & \text{for}\ I_{2}\xrightarrow {\delta_{2}} \phi \\ d\vec{m_{10}}=(0, 0, 0, -1, 0, 0)\ &\text{for} \ V_{2}\xrightarrow {c_{2}} \phi. \end{array} $$

If the rate of the *i*^*th*^ reaction is defined as *a*_*i*_ such that *a*_1_=*β*_1_*TV*_1_,*a*_2_=*β*_2_*TV*_2_, *a*_3_=*k*_1_*E*_1_,*a*_4_=*k*_2_*E*_2_, *a*_5_=*δ*_1_*I*_1_,*a*_6_=*δ*_2_*I*_2_, *a*_7_=*p*_1_*I*_1_,*a*_8_=*p*_2_*I*_2_, *a*_9_=*c*_1_*V*_1_,*a*_10_=*c*_2_*V*_2_, then the probability that the *i*^*th*^ reaction is the next reaction is given by

$$\begin{array}{*{20}l} P_{i}(\vec{m})&=\frac{a_{i}(\vec{m})}{Z(\vec{m})}\\ \text{where}\ Z(\vec{m})&=\sum_{i}^{n_{max}} a_{i}(\vec{m}), \end{array} $$

and *n*_*max*_ is the number of transitions involved in the model and is equal to 10. The time of the next reaction is a random variable with distribution $Z(\vec {m}) \exp (-Z(\vec {m})t)$ with mean $\frac {1}{Z(\vec {m})}$ (according to the Gillespie algorithm). The probability that a simultaneous exposure to both viruses eventually evolves to extinction, or reaches the absorbing state, (0,0,0,0,0,0), from state $\vec {m}$ or the extinction coefficient, $\xi (\vec {m})$, is

1 $$\begin{array}{*{20}l} \xi(\vec{m})&=\sum_{i}P_{i}(\vec{m})\xi(\vec{m}+d\vec{m_{i}}), \vec{m}\neq \vec{0},\\ \xi(\vec{0})&=1\ \text{when} \ \vec{m}= \vec{0}\text{.}\notag \end{array} $$

Substituting the expressions for $P_{i}(\vec {m})$ and $\xi (\vec {m}+d\vec {m_{i}})$ in Eq. (1), the extinction coefficient becomes:

2 $$\begin{array}{*{20}l} \xi(\vec{m})&=\frac{\beta_{1}T{V_{1}}}{Z}\rho^{n_{V_{1}}}_{V_{1}}\rho^{n_{E_{1}}+1}_{E_{1}}\rho^{n_{I_{1}}}_{I_{1}}\rho^{n_{V_{2}}}_{V_{2}}\rho^{n_{E_{2}}}_{E_{2}}\rho^{n_{I_{2}}}_{I_{2}}\\&\quad+\frac{k_{1}{E_{1}}}{Z}\rho^{n_{V_{1}}}_{V_{1}}\rho^{n_{E_{1}}-1}_{E_{1}}\rho^{n_{I_{1}}+1}_{I_{1}}\rho^{n_{V_{2}}}_{V_{2}}\rho^{n_{E_{2}}}_{E_{2}}\rho^{n_{I_{2}}}_{I_{2}}\notag \\&+\frac{p_{1}{I_{1}}}{Z}\rho^{n_{V_{1}}+1}_{V_{1}}\rho^{n_{E_{1}}}_{E_{1}}\rho^{n_{I_{1}}}_{I_{1}}\rho^{n_{V_{2}}}_{V_{2}}\rho^{n_{E_{2}}}_{E_{2}}\rho^{n_{I_{2}}}_{I_{2}}\\&\quad+\frac{\delta_{1}{I_{1}}}{Z}\rho^{n_{V_{1}}}_{V_{1}}\rho^{n_{E_{1}}}_{E_{1}}\rho^{n_{I_{1}}-1}_{I_{1}}\rho^{n_{V_{2}}}_{V_{2}}\rho^{n_{E_{2}}}_{E_{2}}\rho^{n_{I_{2}}}_{I_{2}}\notag \\&+\frac{c_{1}{V_{1}}}{Z}\rho^{n_{V_{1}}-1}_{V_{1}}\rho^{n_{E_{1}}}_{E_{1}}\rho^{n_{I_{1}}}_{I_{1}}\rho^{n_{V_{2}}}_{V_{2}}\rho^{n_{E_{2}}}_{E_{2}}\rho^{n_{I_{2}}}_{I_{2}}\\&\quad+ \frac{\beta_{2}T{V_{2}}}{Z}\rho^{n_{V_{1}}}_{V_{1}}\rho^{n_{E_{1}}}_{E_{1}}\rho^{n_{I_{1}}}_{I_{1}}\rho^{n_{V_{2}}}_{V_{2}}\rho^{n_{E_{2}}+1}_{E_{2}}\rho^{n_{I_{2}}}_{I_{2}}\\&+ \frac{k_{2}n_{E_{2}}}{Z}\rho^{n_{V_{1}}}_{V_{1}}\rho^{n_{E_{1}}}_{E_{1}}\rho^{n_{I_{1}}}_{I_{1}}\rho^{n_{V_{2}}}_{V_{2}}\rho^{n_{E_{2}}-1}_{E_{2}}\rho^{n_{I_{2}}+1}_{I_{2}}\\&\quad+ \frac{p_{2}{I_{2}}}{Z}\rho^{n_{V_{1}}}_{V_{1}}\rho^{n_{E_{1}}}_{E_{1}}\rho^{n_{I_{1}}}_{I_{1}}\rho^{n_{V_{2}}+1}_{V_{2}}\rho^{n_{E_{2}}}_{E_{2}}\rho^{n_{I_{2}}}_{I_{2}} \\&+ \frac{\delta_{2}{I_{2}}}{Z}\rho^{n_{V_{1}}}_{V_{1}}\rho^{n_{E_{1}}}_{E_{1}}\rho^{n_{I_{1}}}_{I_{1}}\rho^{n_{V_{2}}}_{V_{2}}\rho^{n_{E_{2}}}_{E_{2}}\rho^{n_{I_{2}}-1}_{I_{2}}\\&\quad+ \frac{c_{2}{V_{2}}}{Z}\rho^{n_{V_{1}}}_{V_{1}}\rho^{n_{E_{1}}}_{E_{1}}\rho^{n_{I_{1}}}_{I_{1}}\rho^{n_{V_{2}}-1}_{V_{2}}\rho^{n_{E_{2}}}_{E_{2}}\rho^{n_{I_{2}}}_{I_{2}}. \end{array} $$

Although the general solution of this expression is intractable, the CTMC assumption of independent events means that the functional equation of $\xi (\vec {m})$ can be reduced to an algebraic equation. Thus the extinction probability from a given state is the product of the extinction probabilities from each of the constituents of that state [44], so we can write

3 $$\begin{array}{*{20}l} \xi(\vec{m})&=\xi(n_{E_{1}},n_{I_{1}}, n_{V_{1}}, n_{E_{2}}, n_{I_{2}}, n_{V_{2}})\\&\quad=\rho^{n_{V_{1}}}_{V_{1}}\rho^{n_{E_{1}}}_{E_{1}}\rho^{n_{I_{1}}}_{I_{1}}\rho^{n_{V_{2}}}_{V_{2}}\rho^{n_{E_{2}}}_{E_{2}}\rho^{n_{I_{2}}}_{I_{2}}, \end{array} $$

where $\rho ^{n_{V_{1}}}_{V_{1}}$ is the probability that the virus, *V*_1_, initiates a process with $n_{V_{1}}$ number of virus particles that results in extinction. In a similar manner, $\rho ^{n_{E_{1}}}_{E_{1}}, \rho ^{n_{I_{1}}}_{I_{1}}$ and others are probabilities for eclipse cell, *E*_1_, or infected cell, *I*_1_ and so on. Eq. (3) is recognizable as the fixed-point equation $\vec {\varepsilon }= P(\vec {\varepsilon })$, where $\vec {\varepsilon }= [\varepsilon _{1}, \ldots, \varepsilon _{J}]$ and $P(\vec {\varepsilon })$ is the probability generating function of the progeny distributions. Now substituting Eq. (3) in Eq. (2), we get

$$\begin{array}{*{20}l} \rho^{n_{V_{1}}}_{V_{1}}&=\frac{\beta_{1}T}{\beta_{1}T+c_{1}}\rho^{n_{V_{1}}}_{V_{1}}\rho_{E_{1}}+\frac{c_{1}}{\beta_{1}T+c_{1}}\rho^{n_{V_{1}}-1}_{V_{1}}\\ \text{or,}\ \rho_{V_{1}}&=\frac{\beta_{1}T}{\beta_{1}T+c_{1}}\rho_{V_{1}}\rho_{E_{1}}+\frac{c_{1}}{\beta_{1}T+c_{1}}\text{.}\\ \rho^{n_{I_{1}}}_{I_{1}}&=\frac{p_{1}}{p_{1}+\delta_{1}}\rho_{V_{1}}\rho^{n_{I_{1}}}_{I_{1}}+\frac{\delta_{1}}{p_{1}+\delta_{1}}\rho^{n_{I_{1}}-1}_{I_{1}}\\ \text{or,}\ \rho_{I_{1}}&=\frac{p_{1}}{p_{1}+\delta_{1}}\rho_{V_{1}}\rho_{I_{1}}+\frac{\delta_{1}}{p_{1}+\delta_{1}},\\ \rho^{n_{E_{1}}}_{E_{1}}&=\rho^{n_{E_{1}}-1}_{E_{1}}\rho_{I_{1}},\ or \ \rho_{E_{1}}=\rho_{I_{1}}\ \text{and}\\ \rho^{n_{V_{2}}}_{V_{2}}&=\frac{\beta_{2}T}{\beta_{2}T+c_{2}}\rho^{n_{V_{2}}}_{V_{2}}\rho_{E_{2}}+\frac{c_{2}}{\beta_{2}T+c_{2}}\rho^{n_{V_{2}}-1}_{V_{2}}\\ \text{or,}\ \rho_{V_{2}}&=\frac{\beta_{2}T}{\beta_{2}T+c_{2}}\rho_{V_{2}}\rho_{E_{2}}+\frac{c_{2}}{\beta_{2}T+c_{2}},\\ \rho^{n_{I_{2}}}_{I_{2}}&=\frac{p_{2}}{p_{2}+\delta_{2}}\rho_{V_{2}}\rho^{n_{I_{2}}}_{I_{2}}+\frac{\delta_{2}}{p_{2}+\delta_{2}}\rho^{n_{I_{2}}-1}_{I_{2}}\\ \text{or,}\ \rho^{n_{I_{2}}}_{I_{2}}&=\frac{p_{2}}{p_{2}+\delta_{2}}\rho_{V_{2}}\rho_{I_{2}}+\frac{\delta_{2}}{p_{2}+\delta_{2}},\\ \rho^{n_{E_{2}}}_{E_{2}}&=\rho^{n_{E_{2}}-1}_{E_{2}}\rho_{I_{2}}\ or \ \rho_{E_{2}}=\rho_{I_{2}}, \end{array} $$

where $\rho _{V_{i}}$, $\rho _{I_{i}}$ and $\rho _{E_{i}}$ are the extinction probabilities when the processes are initiated with a single virus particle or eclipse cell or infectious cell. Solving for each probability, we get $\rho _{V_{i}}=1$ and $\rho _{V_{i}}= \frac {c_{i}(p_{i}+\delta _{i})}{p_{i}(c_{i}+\beta _{i}T)}$, $\rho _{I_{i}}=1$ and $\rho _{I_{i}}=\frac {\delta _{i}(c_{i}+\beta _{i}T)}{\beta _{i}T(p_{i}+\delta _{i})}$, and $\rho _{E_{i}}=\rho _{I_{i}}$ where *i*=1,2. Since probability has to be less than or equal to 1, we can write the solutions of the extinction probabilities as:

$$\begin{array}{*{20}l} \rho_{V_{1}}&=\text{min}\left(\frac{c_{1}(p_{1}+\delta_{1})}{p_{1}(c_{1}+\beta_{1}T)}, 1\right),\\ \rho{I_{1}}&=\text{min}\left(\frac{\delta_{1}(c_{1}+\beta_{1}T)}{\beta_{1}T(p_{1}+\delta_{1})}, 1\right), \\ \rho_{E_{1}}&=\rho{I_{1}},\\ \rho_{V_{2}}&=\text{min}\left(\frac{c_{2}(p_{2}+\delta_{2})}{p_{2}(c_{2}+\beta_{2}T)}, 1\right),\\ \rho{I_{2}}&=\text{min}\left(\frac{\delta_{2}(c_{2}+\beta_{2}T)}{\beta_{2}T(p_{2}+\delta_{2})}, 1\right),\\ \rho_{E_{2}}&=\rho{I_{2}}\text{.} \end{array} $$

**Probability of virus extinction** Since the extinction of each event is independent, we can write for the probability that both viruses go extinct if the simultaneous infection is initiated with a single virus of each type by the expression $\rho _{V_{1}}\rho _{V_{2}}$,

$$\rho_{V_{1}}\rho_{V_{2}}=\frac{c_{1}(p_{1}+\delta_{1})}{p_{1}(c_{1}+\beta_{1}T_{0})}\frac{c_{2}(p_{2}+\delta_{2})}{p_{2}(c_{2}+\beta_{2}T_{0})}\text{.}  $$

### Stochastic dynamics of identical viruses

While the probability of virus extinction is an important feature of stochastic models, we are also interested in understanding if stochasticity affects the predicted dynamics of coinfections that survive. Previously in our ODE model [27], we found that the virus with the higher growth rate always out-competes the slower growing virus. While ODEs can give us the average behaviors of the coinfection process, in real systems the biological processes are stochastic. The randomness associated with births and deaths during the initial infection process may lead to virus extinction even in an exponentially growing virus population [45]. Yan et al. [34] reported that the invasion of viral infection is dependent on the initial viral dose and growth rate of each virus. Here, we are interested in knowing how the coinfection dynamics change with change in growth rates of each virus. First, we will observe the dynamics of coinfection with identical viruses.

**Fig. 1:**
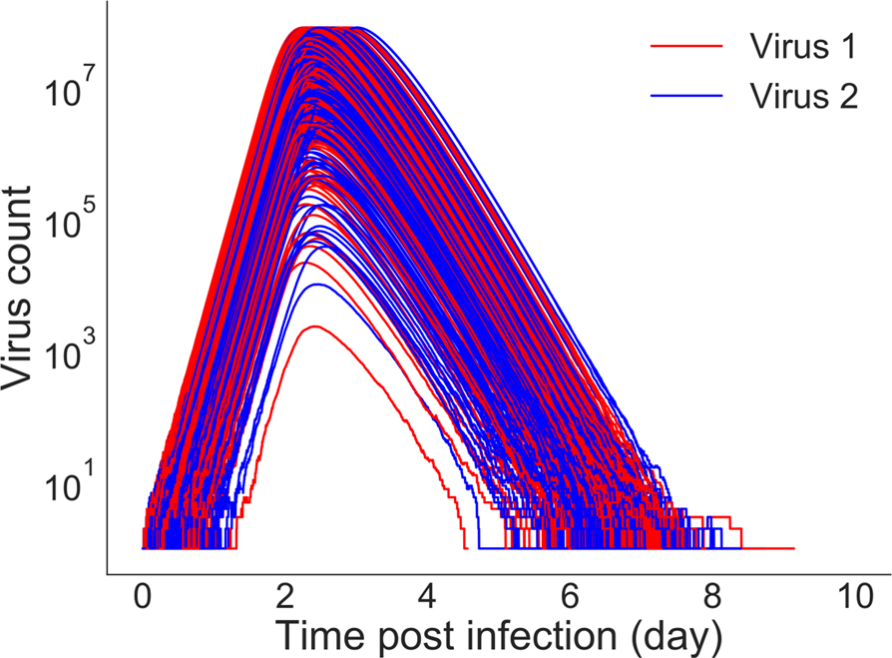
Stochastic trajectories for viruses with the same growth rates. All initial conditions and parameters are also kept equal

Keeping all the initial conditions and transition rates for both viruses equal, we examine the time course of coinfection by plotting number of virus over time. 1000 sample stochastic trajectories of viral load curve for coinfection with identical viruses are shown in Fig. 1. We find that both viruses have peaks above the threshold of detection (100 virions) 88% of the time and 12% of the time one of the viruses experiences extinction. Among 120 (12%) extinctions, virus 1 and virus 2 experience extinction 49 and 65 times out of 1000 simulations, respectively. In another words, there is a 4.9% chance that what begins as a coinfection will result in a single virus infection with virus 2 or 6.5% chance with virus 1.

**Fig. 2:**
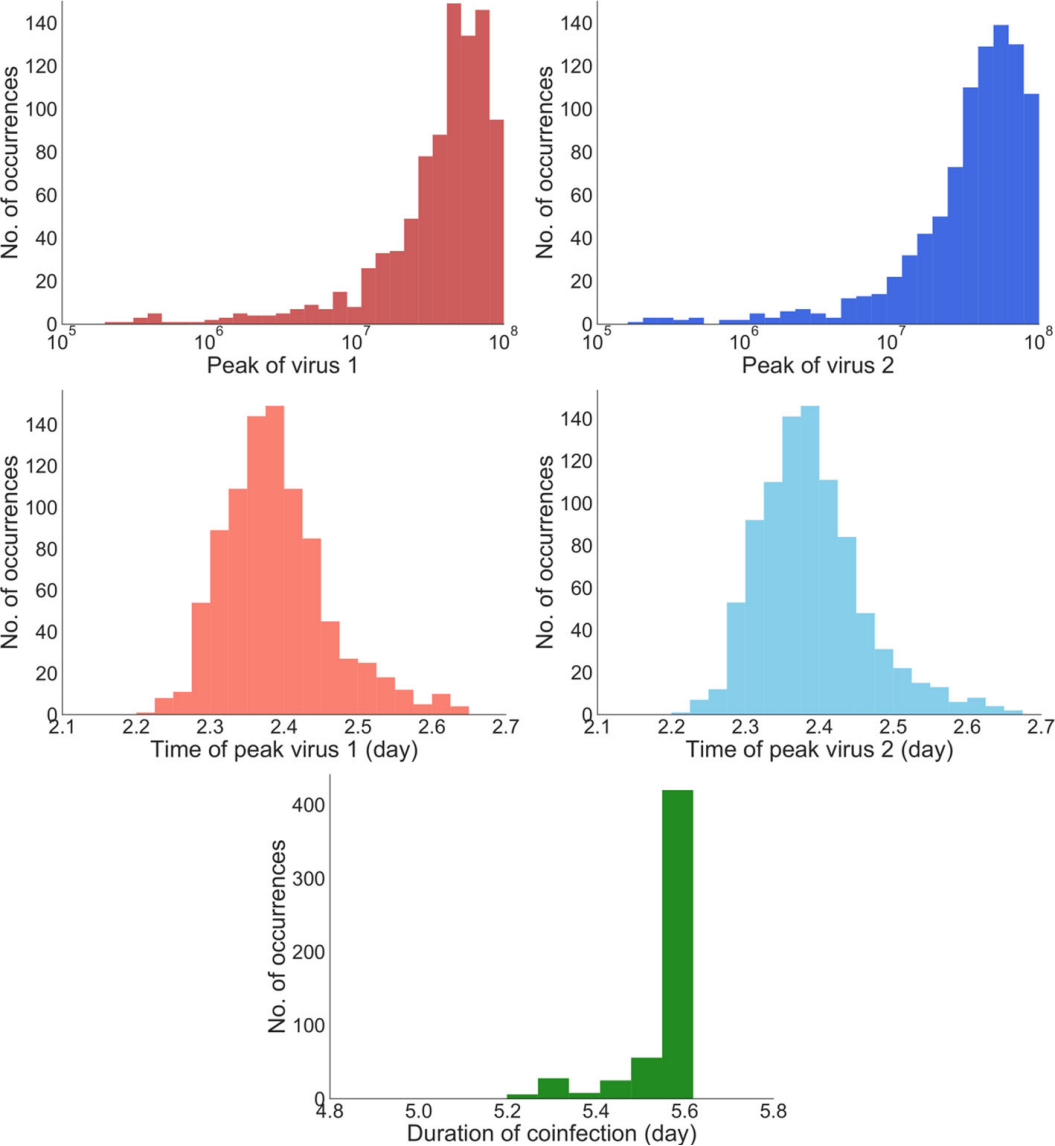
Stochastic dynamics of identical viruses. Distribution of time of peak (top row), peak viral load (middle row) for virus 1 (left column) and virus 2 (right column) and duration of coinfection (bottom row)

The ODE model predicts that when all parameters are equal, both viruses will have the same time course, splitting available target cells equally. In the stochastic model, we find that despite having identical growth rates, one virus out-competes the other virus in particular realizations of the model. Virus 1 has a higher peak viral titer 513 times within 1000 simulations, while virus 2 has the higher peak viral titer 487 times. So while a particular realization of the model will have a clear dominant virus, on average, the viruses are equivalent, in agreement with the ODE model. Additional file 1 includes additional figures examining the distributions when viruses differ. To characterize the viral time course, we calculate peak viral load, time of peak for each virus, as well as duration of coinfection (Fig. 2). The median time of peak for virus 1 is found to be 2.374 ±0.64 days and for virus 2, it is 2.375 ±0.65 days. The median of peak viral load for virus 1 and 2 are (4.0 ±2.6) ×10^7^ and (4.1 ±2.6) ×10^7^, respectively. From the distributions (Fig. 2), we see that even if the viruses behave differently for a particular model realization, on average, they tend to behave identically. Finally, the distribution of coinfection duration is given in Fig. 2 where the median coinfection duration is found to be 5.730 ±0.059 days. Despite fluctuations in the time course of each virus, the coinfection duration does not vary much.

### Stochastic dynamics for different viruses

**Fig. 3:**
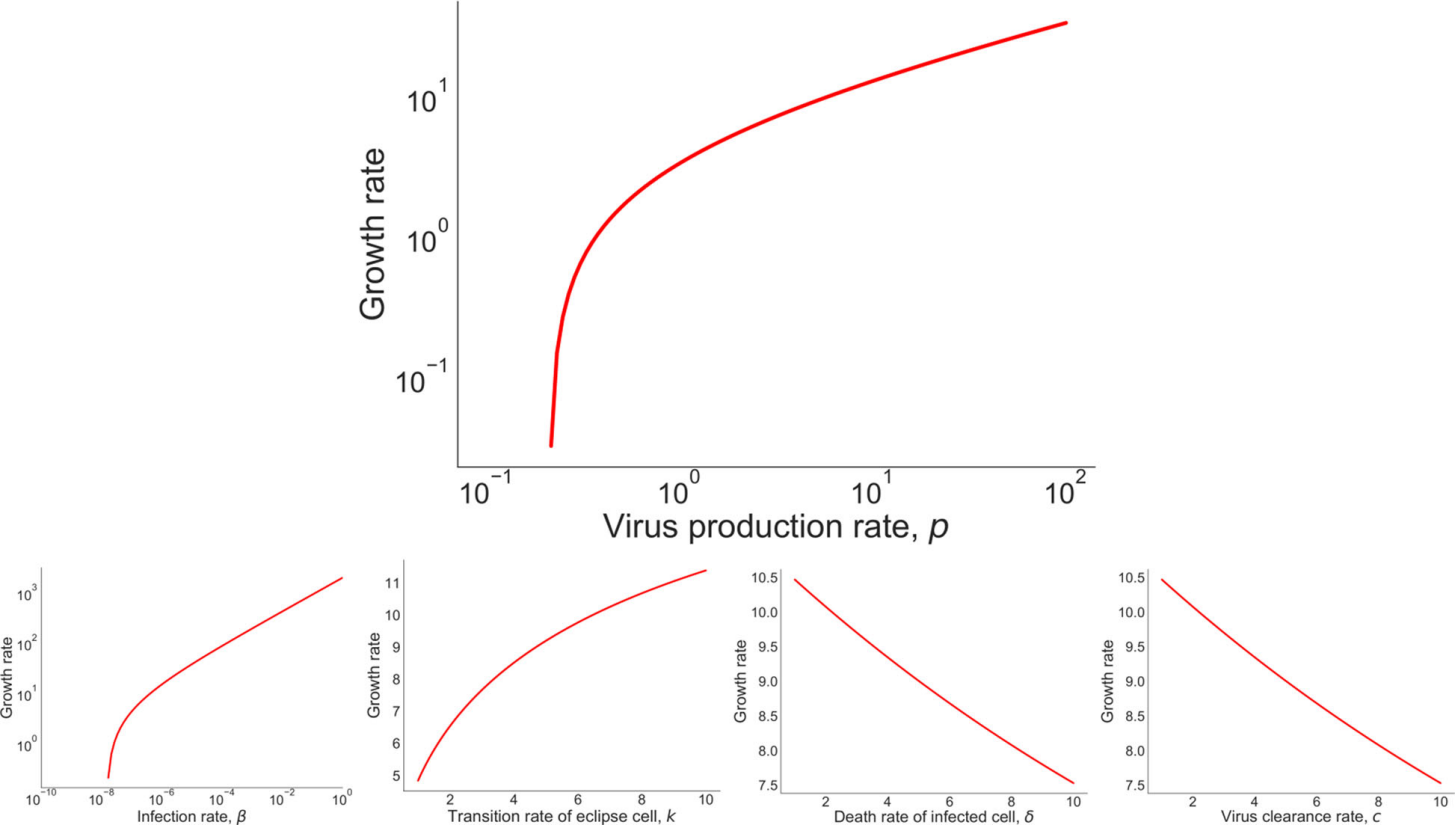
Variation of growth rate with respect to model parameters

Since growth rate determines which virus is the stronger competitor [27], we investigate how differences in growth rate between the two viruses changes stochastic infections. Unfortunately, growth rate is not a parameter in the model, so we need to determine which model parameter(s) to change to systematically vary the growth rate. We use the expression for growth rate derived by Smith et al. [46] and determine how growth rate depends on different model parameters (Fig. 3). We find that growth rate varies approximately linearly with virus production rate, *p*, over a large range of *p* (*p*>1), so we will systematically alter *p* for one virus to alter its growth rate.

**Fig. 4:**
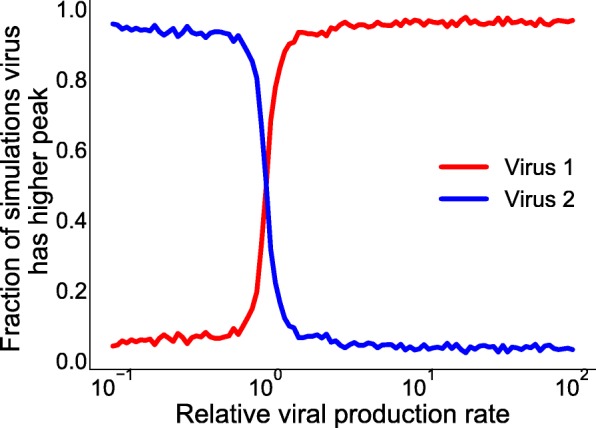
Number of times one virus has a higher peak viral titer than the other virus. Growth rate is varied by varying the relative viral production rate, $r=\frac {p_{1}}{p_{2}}$

For ease of interpretation, we define the relative viral production rate $r = \frac {p_{1}}{p_{2}}$. We first examine how competition between the viruses changes as the relative growth rates change. Here variation is introduced for virus 1 keeping virus 2 fixed for a range, *r*=1×10^−1^×10^2^. We count the number of times, out of 1000 simulated infections, a particular virus has a higher viral titer peak than the other virus. The results are shown in Fig. 4. When the viruses have identical growth rates, there is a 50% chance that a particular virus will have the higher peak titer, as seen in the previous section. The probability of having a higher peak viral load increases rapidly as the production rate of a virus increases, reaching 90% with a less than 2-fold change in viral production. Note that the probability of having the higher peak viral titer never quite reaches 100%, even when there are large differences in growth rate. This indicates that early stochastic events can significantly alter the time course of the infection.

**Fig. 5:**
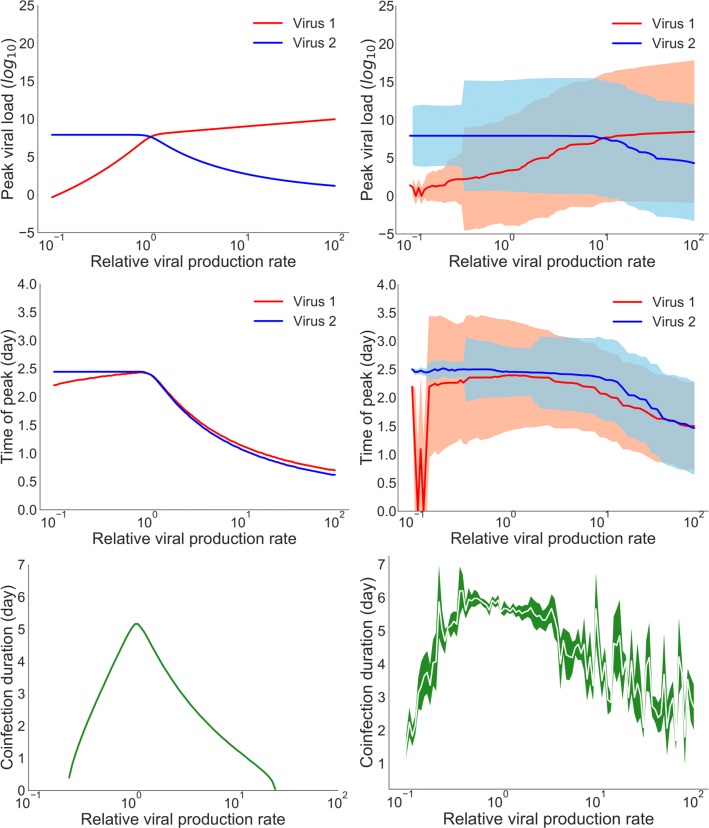
Comparison of the ODE and the CTMC model infection dynamics. Variation in peak viral load (top row), time of viral peak (center row), and duration of coinfection (bottom row) as a function of relative viral production in the ODE model (left column) and in the stochastic model (right column). For the stochastic model, solid lines indicate the median of 1000 simulations, with shaded areas indicating the standard deviation

In Fig. 5, we compare coinfection dynamics for the ODE and CTMC models, looking at peak viral load, time of viral peak, and coinfection duration. ODEs predict that if the growth rate of one virus is higher than the other it will *always* have a higher peak viral load (Fig. 5 (top left)). For the CTMC model, the transition from one virus dominating to the other dominating is not as sharp. Unlike the predictions of ODEs, the CTMC allows for the slower growing virus to dominate infection dynamics. In fact, the median peak viral loads for virus 1 and virus 2 cross closer to a relative viral production rate of 10^1^ rather than 10^0^ as seen in the ODE model. Stochastic variability in the peak viral load (as indicated by the shaded area) for both viruses overlaps for a wide range of relative viral production, indicating that the viruses can have similar peak viral loads.

The time of viral peak also shows some differences between the ODE and CTMC models. For the ODE model, the time of viral peak is similar for both viruses when the relative viral production rate is greater than 10^0^, although the time of peak decreases as the relative viral production rate increases. This is because the viral production rate of virus 1 is increased over its baseline value causing an earlier time of peak. This drives the earlier time of peak of virus 2, which is the weaker competitor in this case. The decline in time of viral peak is not as sharp in the CTMC model since stochasticity can temper the effect of the increased production rate of virus 2 by allowing virus 1 to still have an opportunity to infect some cells.

Finally, we compare the predicted duration of coinfection variation for ODE and stochastic models (Fig. 5 (bottom row)). Viruses do not coexist for more than about a week in either model. The longest coinfection durations are seen, for both models, when the two viruses have the same growth rates. This is because the faster growing virus out-competes the slower growing virus leading to short infections for the slower growing virus.

**Fig. 6:**
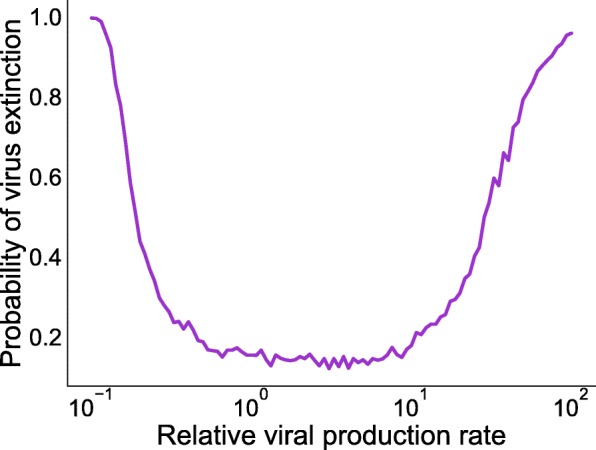
Probability of virus extinction with respect to varying relative production rate. Extinction probability is simulated for the case when the coinfection is initiated with a single copy of each virus and the virus detection limit is set at 100 virions

One feature of viral infections that cannot be captured by ODE models is extinction of the infection. Therefore, we simulate the probability of virus extinction, defined as the fraction of times when one virus does not grow above the virus detection limit (detection limit is equal to 100 virus particles), when the coinfection is initiated with a single virus of each type (Fig. 6). Note that this is slightly different than the definition for the probability of extinction calculated in “Derivation of extinction coefficient” section which requires that virus, along with infectious and eclipse cells, all go to zero. The probability of both viruses growing to detectable levels is highest for viruses with similar relative production rates. When relative viral production rates are very different (about 10–100 fold difference), there is a high probability that one virus becomes extinct. When the viruses have very different production rates, the virus with a higher production rate will out-compete the one with a low production rate driving it to extinction. However, since one virus (in this case virus 1) experiences decreased production rate from the base value but initiates infection with the same amount of virus, the probability of extinction reaches close to 100% in a quicker manner for lower relative production rate than that of the higher relative rates.

## Discussion

The dynamics of coinfection were previously modeled deterministically in several studies [24, 25, 29]. However, ODE models do not capture the very earliest dynamics of infection where stochastic effects may play an important role. The stochastic model presented here indicates that stochastic effects can dramatically alter the time course of the infection. Our previous ODE coinfection model [27] could not distinguish between two identical/similar viruses, as the predicted time courses are identical. Simulations of the stochastic model, however, indicate that for a particular realization of the model, two identical viruses can have very different time courses, with ∼12% of infections initiated with two viruses resulting in infections with only one detectable virus. When viruses have different growth rates, the ODE model predicts that the virus with the higher growth rate will have a higher peak viral titer. This is not the case for the CTMC where early stochastic effects can allow a slower growing virus to infect more target cells than the faster growing virus, giving the slower virus a competitive advantage that continues over the course of the infection.

The ODE coinfection model resulted in a simple rule for determining which virus would be dominant in a coinfection — the virus with a higher growth rate. Replication of the slower-growing virus is suppressed due to lack of accessibility to target cells. This simple rule suggests that we can easily determine which viruses will be suppressed in coinfections. For example, application of the ODE model to several respiratory viruses indicated that parainfluenza virus (PIV) replication is substantially reduced during coinfection with other respiratory viruses [27], suggesting that it should be difficult to detect PIV in coinfections. However, PIV is detected in coinfections from 30–80% of the time [15, 47–50]. Some of this unexpectedly high detection rate might be due to stochasticity. PIV detection in coinfection is, however, lower than what is observed for two identical viruses as described in the previous paragraph. The slow growth rate of PIV means that most viruses will out-compete PIV more often than viruses with identical growth rates.

Stochasticity also impacts our ability to use viral interference as a possible mechanism for treating or preventing more serious infections. If we cannot guarantee that a fast-growing virus will suppress growth of a slow-growing virus, then this strategy might be risky. For example, some have suggested using defective interfering particles (DIPs) as a possible method for blocking infections [51–55]. DIPs cannot replicate on their own, but have a high growth rate when fully-functioning virus is present. Our results indicate that even when there is a large difference in the viral growth rate, there is a non-zero probability that the slower-growing virus (in this case the fully-functional virus) will rise to a higher peak than the faster growing virus, suggesting that use of DIPs for treatment will not be completely effective.

While our extension of the simple coinfection model has provided insight into how stochasticity might affect coinfections, this simple model does not capture all biological processes during the infection. More complex ODE models that include cell regeneration [29] and superinfection [30] have been proposed and reproduce a broader range of behaviors observed during viral coinfections. Stochastic versions of these models can also be developed in the future to examine how stochasticity affects behaviors such as chronic coinfections. Other limitations include the lack of an explicit immune response, which will likely increase the probability of extinction of the coinfection [34], and the inclusion of realistic delays to account for intracellular replication [56]. Despite these shortcomings, this stochastic implementation of a viral coinfection model has shown the extent of variability in the time course of coinfections when stochasticity is introduced.

## Conclusions

While ODE models are useful for giving a broad picture of possible dynamical behaviors of infection, in reality each infection is distinct with disease outcome dependent on early stochastic events. This is particular important when considering interactions between viruses during coinfection since stochasticity can drive one or both viruses to extinction before the infection has time to take hold. Our models show that for viral coinfections, this sometimes leads to a less fit virus out-competing a more fit virus.

## Methods

### Continuous-time Markov chain model

**Fig. 7:**
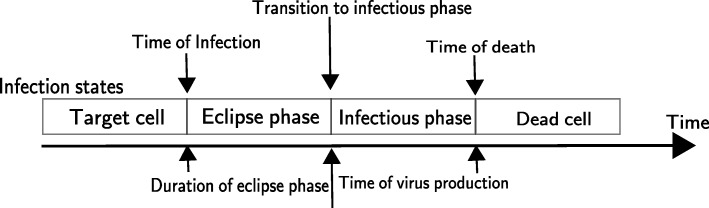
Stochastic states and their transitions during coinfection. All infection states are the same for each virus and viruses share the same pool of target cells

The previously [27] proposed ODE coinfection model considers the mean concentrations of viruses and cells in a large population. Here, we formulate the probabilistic counterpart of the ODE model, a time-homogeneous CTMC model of two competing viruses with particular account for stochastic effects in the early infection processes. This model considers variability in each viral replication event (for example, infectivity of target cells, transition to eclipse phase, activation of infectious phase and its lifespan, virus production and virus clearance) and takes values on a set of states collectively known as the state space *Ω*. The states of the full system are defined as $\vec {m}=(n_{T}, n_{E_{1}}, n_{I_{1}}, n_{V_{1}}, n_{E_{2}}, n_{I_{2}}, n_{V_{2}})$ where the state vectors denote the integer numbers of target cells, eclipse cells, infected cells, virions for virus 1 and 2 respectively. The states are discrete, and the stochastic process is time-homogeneous. The CTMC model that we implement is similar to that of Pearson et al. [33]. Figure 7 illustrates the model diagram. The model is

$$\begin{array}{*{20}l} T+V_{1}\xrightarrow {\beta_{1}} E_{1} && T+V_{2}\xrightarrow {\beta_{2}} E_{2}\\ E_{1}\xrightarrow {k_{1}} I_{1} && E_{2}\xrightarrow {k_{2}} I_{2}\\ I_{1}\xrightarrow {p_{1}} V_{1} && I_{2}\xrightarrow {p_{2}} V_{2}\\ I_{1}\xrightarrow {\delta_{1}} \emptyset && I_{2}\xrightarrow {\delta_{2}} \emptyset \\ V_{1}\xrightarrow {c_{1}} \emptyset && V_{2}\xrightarrow {c_{2}} \emptyset, \\ \end{array} $$

where *T* is the number of susceptible target cells, *E*_1_ and *E*_2_ are the number of eclipse cells or non-infectious infected cells, *I*_1_ and *I*_2_ are the number of active infectious cells and *V*_1_ and *V*_2_ are the number of virus particles. Viruses of each type infect the target cells, which are limited, at infection rates, *β*_1_ and *β*_2_. Each type of infected cell transitions into eclipse phases where viruses undertake intracellular processes for progeny virus production. After the time durations of $\frac {1}{k_{1}}$ and $\frac {1}{k_{2}}$, eclipse cells become productive infectious cells, *I*_1_ and *I*_2_, which produce viruses at production rates *p*_1_ and *p*_2_ over their lifespans of $\frac {1}{\delta _{1}}$ and $\frac {1}{\delta _{2}}$ respectively. While viruses are being produced by the infectious cells, some of the cell free viruses are being cleared with clearance rates *c*_1_ and *c*_2_. Thus the number of transitions involved in the model is 10 with respective transition rates (propensities) defined in Table 1. In our model assumption, we have ignored virus particle loss due to absorption into the cells since this amount is negligible compared to free virus particles produced. Finally, no specific immune interactions are considered in this model.

**Table 1:** State transitions and propensities for the CTMC coinfection model

Description	Transition	Propensity
Infection by *V*_1_	*T*→*T*−1,*E*_1_→*E*_1_+1	*β*_1_ *T* *V*_1_
Infection by *V*_2_	*T*→*T*−1,*E*_2_→*E*_2_+1	*β*_2_ *T* *V*_2_
Infection to infectious	*E*_1_→*E*_1_−1,*I*_1_⇒*I*_1_+1	*k*_1_ *E*_1_
Infection to infectious	*E*_2_→*E*_2_−1,*I*_2_→*I*_2_+1	*k*_2_ *E*_2_
Death of *I*_1_	*I*_1_→*I*_1_−1	*δ*_1_ *I*_1_
Death of *I*_2_	*I*_2_→*I*_2_−1	*δ*_2_ *I*_2_
Production of *V*_1_	*V*_1_→*V*_1_+1	*p*_1_ *I*_1_
Production of *V*_2_	*V*_2_→*V*_2_+1	*p*_2_ *I*_2_
Decay of *V*_1_	*V*_1_→*V*_1_−1	*c*_1_ *V*_1_
Decay of *V*_1_	*V*_2_→*V*_2_−1	*c*_2_ *V*_2_

It has been shown that, the stochastic representations of chemical reactions converge to the differential equations as the number of particles goes to infinity when we can assume that the probability of a reaction depends on the density of the reactants [57–59]. We make a similar assumption for the “reactions” involved in viral replication where infection of a cell, for example, depends on the density of both cells and virus. Since biological processes, particularly at the microscopic level, are really a series of chemical reactions, there is an inherent stochasticity to the system that is not simply averaged out because we are not specifically considering the detailed chemical reactions in the model. For instance, the infection of a cell in this model includes binding of the virus to the cell receptor, fusion of the virus with the cell membrane, and opening of the virus membrane to release the contents, among other steps. These are all chemical reactions that can be assumed to occur with probability proportional to density of the reacting chemicals. It seems reasonable then to assume that the overall infection process is also dependent on the density of the larger entities (viruses and cells) that contain these chemicals and we can expect a similar convergence of the Markov chain to the differential equation when there are large numbers of viruses and cells.

### Stochastic simulation algorithm

The direct method popularized by Gillespie [60], in general, is used for solving trajectories of time-homogeneous CTMC models. Bartlett [61] first applied this method for epidemic modeling of measles. However, since the computing time of the direct method scales linearly with the initial number of target population [34], the direct method becomes infeasible to simulate viral infection models with realistic number of target cells, i.e. of order 1×10^8^. Due to the increased simulation efficiency with some accuracy trade off, Gillespie tau-leap algorithms are getting more attention. In the tau-leap method a small time interval is chosen such that the number of times each transition occurs in this interval is drawn from a Poisson distribution with mean equal to the expected number of transitions during that interval. The time step is fixed for the most basic tau-leap method. However, the time step should be small enough that the rate at which transitions occur remains approximately constant during the fixed interval. For numerical implementation of the CTMC model, we use the Gillespie tau-leaping method with time step equal to 10^−3^ day. The transitions involved in the stochastic process and their rates are summarized in Table 1. The parameter values for numerical simulation are taken from [19] and are given in Table 2. Viral load is usually measured as concentration with units such as TCID_50_/mL, PFU/mL or EID_50_/mL rather than as the total number of virus in the host, while the CTMC model uses discrete values for each of its state variables. In order to convert viral concentration measurements to the number of infectious virus particles, studies have used a conversion factor, *α* [31, 62, 63]. Although there is no standard value for *α*, previous estimates suggest that 1 TCID_50_/mL of nasal wash corresponds to 1×10^2^−1×10^5^ [62] or 3×10^4^−3×10^5^[63] virus particles at the site of infection. So we take *α* equal to 1×10^2^ to convert the concentrations of virus into numbers of virus particles according to the method mentioned in [31].

**Table 2:** Parameter values for the CTMC coinfection model

Parameter	Value ^*a*^	Units
*β*	3.2×10^−5^	cell ^−1^ (TCID_50_/mL) ^−1^ d ^−1^
*k*	4.6	d ^−1^
*δ*	5.2	d ^−1^
*p*	4.6×10^−2^	TCID_50_/mL d ^−1^
*c*	5.2	d ^−1^
*T*_0_	4.0×10^8^	cell
*V*_0_	7.5×10^−2^	TCID_50_/mL
*α*	1×10^2^	—

^*a*^Parameter values are taken from [19] for influenza A virus

## Additional file

Additional file1 Pdf file containing additional figures. (PDF 26,531 kb)

## Abbreviations

AdV: Adenovirus
CoV: Coronavirus
CTMC: Continuous time Markov chain
DIP: Defective interfering particle
EID: Egg infectious dose
hBoV: Human bocavirus
hEV: Human enterovirus
HIV: Human immunodeficiency virus
hMPV: Human metapneumovirus
hRV: Human rhinovirus
IAV: Influenza A virus
IBV: Influenza B virus
ILI: Influenza-like illness
ODE: Ordinary differential equation
PFU: Plaque forming unit
PIV: Parainfluenza virus
RSV: Respiratory syncytial virus
TCID: Tissue culture infectious dose
