# Supplementary material for: Effect of stochasticity on coinfection dynamics of respiratory viruses
Source: BMC Bioinformatics. 2019 Apr 16;20:191. doi: 10.1186/s12859-019-2793-6 (PMC6469119; doi:10.1186/s12859-019-2793-6)
Supplement: Supplementary file 1 — Pdf file containing additional figures. (PDF 26,531 kb) [file 12859_2019_2793_MOESM1_ESM.pdf]

Supporting information for the article "Effect of stochasticity on coinfection dynamics of respiratory viruses"

Lubna Pinky[1,2], Gilberto González-Parra[3] and Hana M. Dobrovolny[1,*]

[1] Department of Physics & Astronomy, Texas Christian University, Fort Worth, TX, USA
[2] Department of Pediatrics, University of Tennessee Health Science Center, Memphis, TN, USA [3] Department of Mathematics, New Mexico Tech, Socorro, New Mexico, USA
[*] Corresponding author, Department of Physics & Astronomy, Texas Christian University, 2800 S. University Dr., Fort Worth, TX, 76109, USA.
E-mail address: `h.dobrovolny@tcu.edu`

1 Stochastic dynamics of different viruses

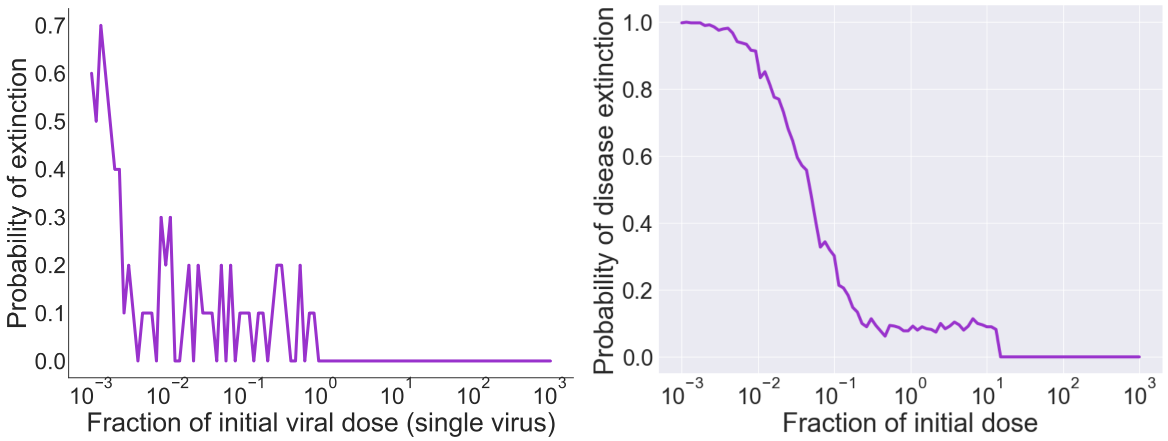

Figure 1: Probability of virus extinction with respect to varying initial viral dose for a single virus infection (left) and coinfection (right). Extinction probability is simulated for the case when the coinfection is initiated with single copy of each virus and virus detection limit is considered 100 virus.

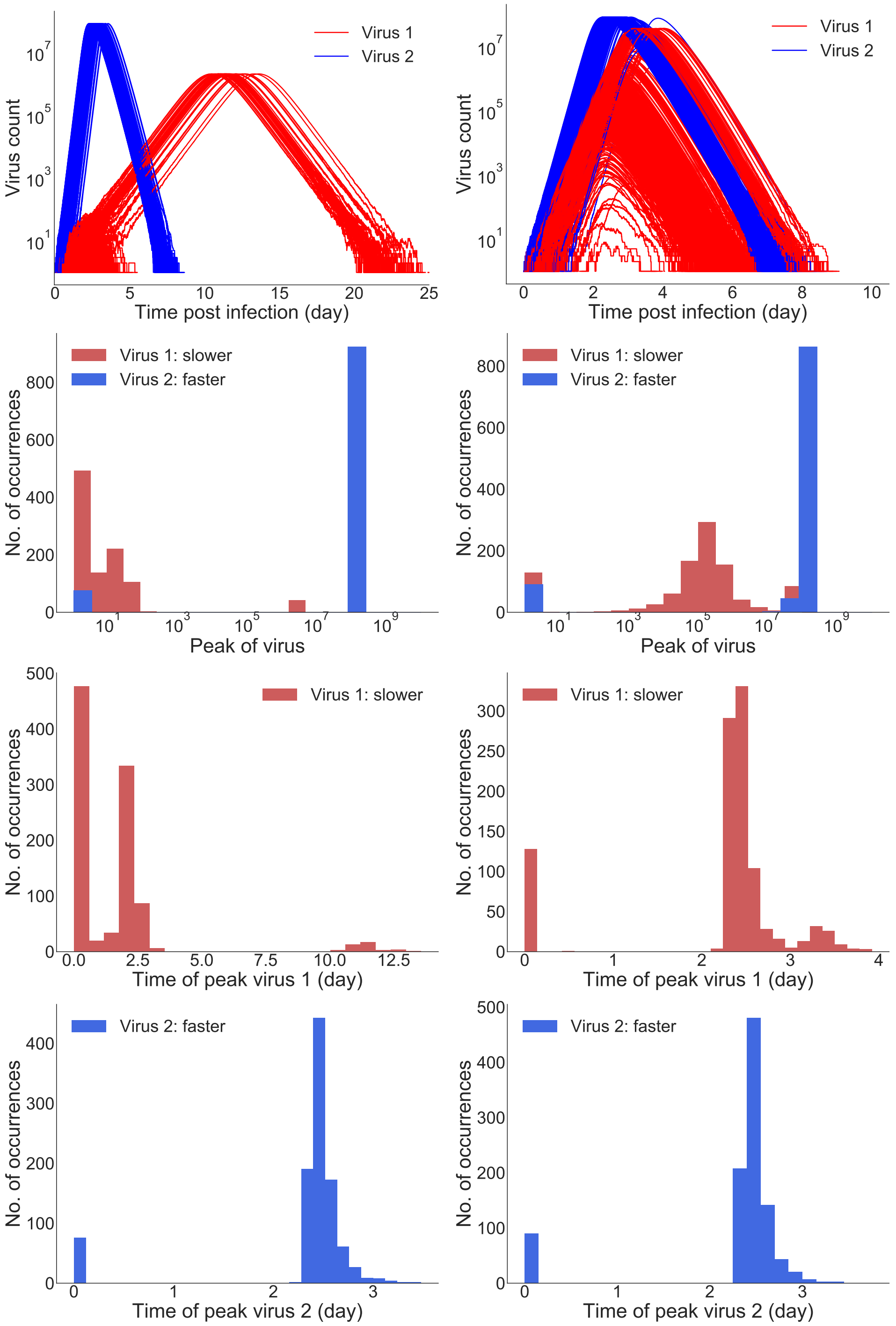

Figure 2: Stochastic dynamics of different viruses. Viruses are different with respect to growth rates (i.e. production rates). Left column shows when virus 1 is 1×10^{-1} times and right column shows when 5×10^{-1} times slower than virus 2, respectively. Stochastic viral load trajectories are shown in top row. Distribution of peak viral loads and time of peak virus 1 and peak virus 2 are shown in second row, third row and bottom row, respectively.

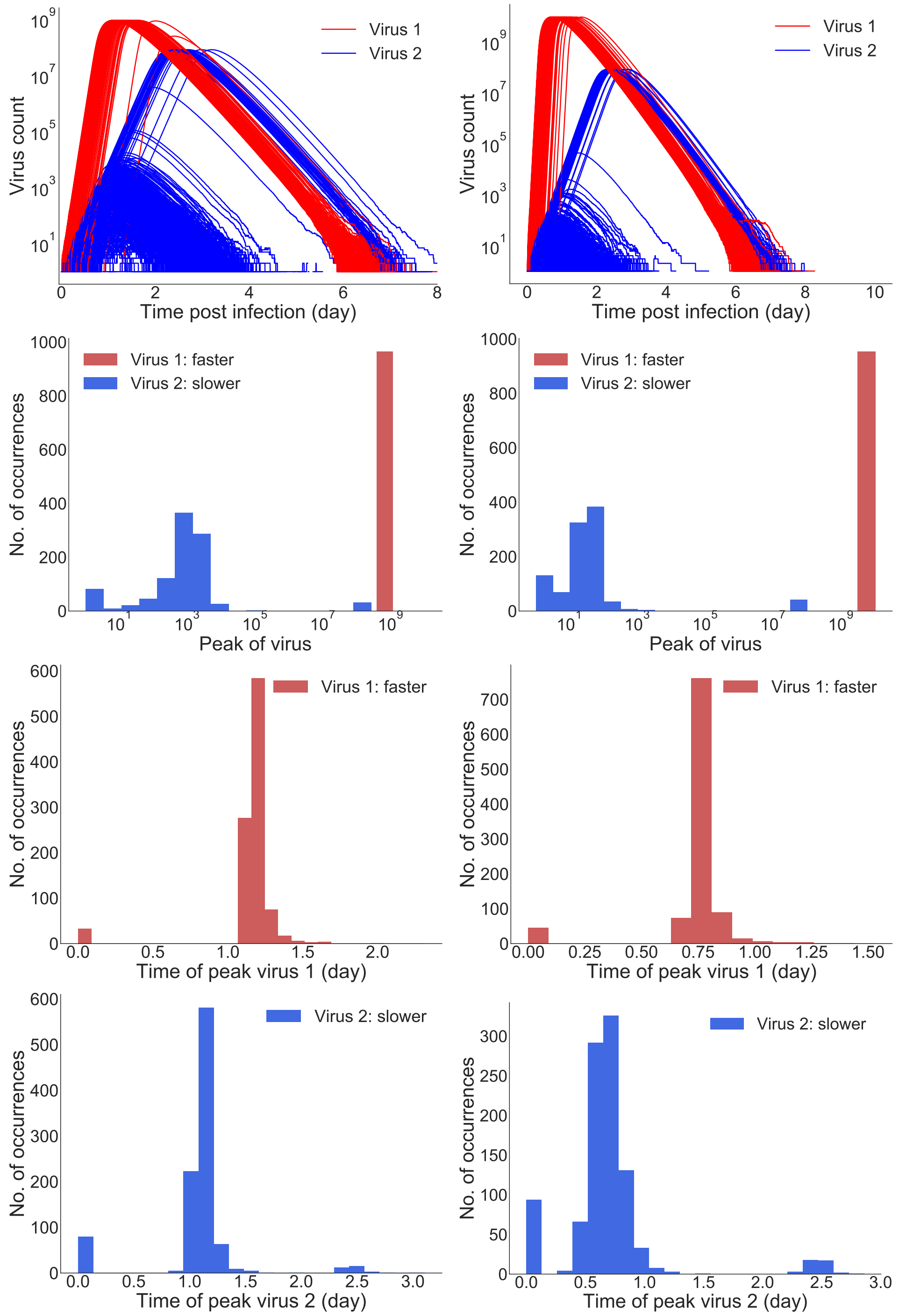

Figure 3: Stochastic dynamics of different viruses. Viruses are different with respect to growth rates (i.e. production rates). Left column shows when virus 1 is 1×10^1 times faster than virus 2 and right column shows when virus 1 is more faster (1×10^2 times) than virus 2. Stochastic viral load trajectories are shown in top row. Distribution of peak viral loads and time of peak virus 1 and peak virus 2 are shown in second row, third row and bottom row, respectively.
